# IRAPs in Combination with Highly Informative ISSRs Confer Effective Potentials for Genetic Diversity and Fidelity Assessment in *Rhododendron*

**DOI:** 10.3390/ijms24086902

**Published:** 2023-04-07

**Authors:** Sulin Wen, Hong Zhao, Manying Zhang, Guang Qiao, Xiaohui Shen

**Affiliations:** 1 School of Design, Shanghai Jiao Tong University, Shanghai 200240, China; 17813209312@163.com; 2 Key Laboratory of Mountain Plant Resources Protection and Germplasm Innovation (Ministry of Education), Guizhou University, Guiyang 550025, China; zhaohonggzu@163.com (H.Z.); my15734445278@163.com (M.Z.)

**Keywords:** genetic fidelity, genetic variation, IRAP, ISSR, polymorphism, *Rhododendron*

## Abstract

The species belonging to the *Rhododendron* genus are well-known for their colorful corolla. Molecular marker systems have the potential to elucidate genetic diversity as well as to assess genetic fidelity in rhododendrons. In the present study, the reverse transcription domains of long terminal repeat retrotransposons were cloned from rhododendrons and used to develop an inter-retrotransposon amplified polymorphism (IRAP) marker system. Subsequently, 198 polymorphic loci were generated from the IRAP and inter-simple sequence repeat (ISSR) markers, of which 119 were derived from the IRAP markers. It was shown that in rhododendrons, IRAP markers were superior to the ISSRs in some polymorphic parameters, such as the average number of polymorphic loci (14.88 versus 13.17). The combination of the IRAP and ISSR systems was more discriminative for detecting 46 rhododendron accessions than each of the systems on their own. Furthermore, IRAP markers demonstrated more efficiency in genetic fidelity detection of in-vitro-grown *R. bailiense* Y.P.Ma, C.Q.Zhang and D.F.Chamb, an endangered species recently recorded in Guizhzhou Province, China. The available evidence revealed the distinct properties of IRAP and ISSR markers in the rhododendron-associated applications, and highlighted the availability of highly informative ISSR and IRAP markers in the evaluation of genetic diversity and genetic fidelity of rhododendrons, which may facilitate preservation and genetic breeding of rhododendron plants.

## 1. Introduction

*Rhododendron* L. consists of a category of species with a significant horticultural importance in the family Ericaceae. It is also renowned as a world-famous alpine flower for its various ecological varieties and vibrant hues. To date, more than 1000 species of wild *Rhododendron* spp. have been reported, mainly in East Asia, Southeast Asia and the Himalayas [1]. In China, *Rhododendron* is the largest genus of woody plants, with approximately 600 species, including many species described after the Flora of China was published [2]. Southwest to central China is the possible origin and principal distribution area of *Rhododendron* [3]. Numerous endemic *Rhododendron* species have been discovered in Guizhou Province of China so far, where wild *Rhododendron* resources are abundant [4]. Among them, the core area of the Baili-Dujuan Nature Reserve (BDNR) in Bijie City, Guizhou Province, China, stretches over 125.8 square kilometers and contains 64 species from five *Rhododendron* subgenera [5], and contains by far the largest, most diverse and best-preserved primitive rhododendron forest in China, and has been labeled as the “*Rhododendron* Kingdom” [6]. Particularly, BDNR possesses a few locally unique species, such as *R. bailiense* Y.P.Ma, C.Q.Zhang and D.F.Chamb [5], which is an endangered species that has been just discovered there and is on the brink of extinction [5]. Characterization of genetic diversity and genotyping the *Rhododendron* germplasms may substantially facilitate the preservation of *Rhododendron* species.

With respect to the importance of rhododendron plants and the scarcity of some varieties, in vitro culture has intensively received increasing attention [7]. In vitro propagation provides a technical attempt for the germplasm preservation of *Rhododendron*, and the studies have been carried out on a variety of germplasms so far. Wei et al. (2018), for example, induced axillary shoots from two-node explants of *R. fortunei* in vitro and developed the most appropriate conditions for micropropagation [7]. Nowakowska et al. (2022) cultured *Rhododendron* ‘Kazimierz Odnowiciel’ in vitro and demonstrated the effect of cytokinins on shoot proliferation and genetic stability of plants [8]. However, the in vitro propagation of many endangered *Rhododendron* species, e.g., *R. bailiense*, has not yet been established so far.

Retrotransposons are important and peculiar genetic elements derived from ancient retrovirus insertions inside the plant genome, whose ability to replicate inside the genome greatly contributes to the occurrence of genetic variation and the increase in genome size. Based on the sequence characters in the genome, several retrotransposon-based marker systems have been established [9], with the inter-retrotransposon amplified polymorphism (IRAP) being the most prominent. LTR retrotransposon-associated IRAP is a powerful marker because of its ability to detect insertion polymorphisms through amplification of the portion of the DNA fragment between double retrotransposons [10]. Due to its simplicity, IRAP technology has been widely employed to detect genetic variation as well as to elucidate the genetic diversity of plant species [11]. For example, IRAP markers were able to distinguish between perennial and annual sunflower (*Helianthus* genus) species, and the diversity identified by IRAP was reduced in domesticated sunflower accessions [12]. IRAPs were also suitable for identifying the genetic diversity of *Phyllostachys*, a genus of Asian bamboo [13]. However, there has not yet been any studies on IRAP markers in rhododendron.

The objective of this study was to develop a molecular marker system to detect the genetic diversity as well as the genetic fidelity/variation of germplasms in rhododendron. This is primarily achieved by (1) cloning and sequencing the LTR retrotransposon sequences in rhododendron, and developing IRAP markers based on these sequences; (2) evaluating the genetic diversity of 46 rhododendron accessions based on the combination of IRAPs and ISSRs; and (3) detecting the genetic fidelity of the in vitro cultures of *R. bailiense*, a very endangered species, using the above-mentioned marker system. These results highlight the availability of highly informative ISSR and IRAP markers in the evaluation of genetic diversity and genetic fidelity of rhododendrons, which may facilitate the preservation and genetic breeding in rhododendron plants.

## 2. Results

### 2.1. Identification of Polymorphic IRAP Markers

The amplified band fragments of the reverse transcription domain (RT domain) of TY1-*Copia* and TY3-*Gypsy* in *R. delavayi* were obtained using PCR amplification. Their sizes were approximately 250 bp and 400 bp, respectively (Figure S2). The amplified bands were purified for DNA fragment cloning. Approximately 60 single clones from each superfamily were randomly selected for sequencing. A total of 23 independent sequences for TY1-*Copia* were obtained after removing repetitions, with a length of 234–245 bp and an average GC content of 44.78% (Table S2). The generated TY1-*Copia* can be divided into four main clades (Figure S3A), among which group I covers the most TY1-*Copia*, reaching 15.

For TY3-*Gypsy*, a total of 23 independent sequences were obtained after removing repeats, with a length of 348–503 bp and an average GC content of 43.14% (Table S2). The sequence of TY3-*Gypsy* demonstrated higher heterogeneity and might be divided into seven clades (Figure S3B). The clade with the largest number of TY3-*Gypsy* which included group I and group IV, had only four TY3-*Gypsy*. According to the identified RT domain sequences of TY1-*Copia* and TY3-*Gypsy*, we designed IRAP primers at appropriate positions (Table S2) for subsequent experiments.

The annealing temperature highly affects the amplification polymorphism of molecular markers. Thus, it is necessary to screen the appropriate annealing temperature of IRAP markers. Many IRAP markers of TY1-*Copia* showed good polymorphism (Figure S4), such as LTR1–7 (56 °C), LTR1–10 (59 °C), LTR1–11 (59 °C), LTR1–12 (59 °C), LTR1–15 (55 °C), LTR1–16 (55 °C), LTR1–17 (56 °C), etc., and then we obtained their optimum annealing temperature through the above screening. However, the IRAP molecular markers designed by TY3-*Gypsy* generally failed to show good amplification polymorphism (Figure S4), and only one IRAP primer was screened, LTR3–21 (57 °C).

### 2.2. Informative Identification of IRAP and ISSR Markers

**Figure 1:**
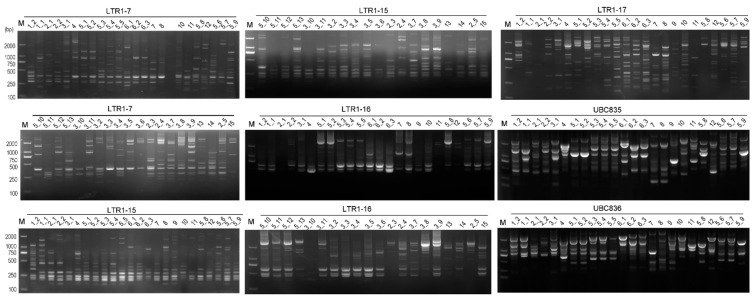
Electrophoresis profiles of the rhododendron accessions from BDNR as revealed by inter-retrotransposon amplified polymorphism (IRAP) and inter-simple sequence repeat (ISSR). The lanes corresponding to the accessions (Table S1) are marked on the top of the gel map. The characters on the horizontal line are the IDs of IRAP or ISSR markers. Lanes that did not exhibit bands were retested and the binary data obtained were incorporated into subsequent analyses. M, D2000 DNA marker (Tiangen Biological Company, Beijing, China).

Hong et al. (2012) found 11 ISSR polymorphic primers suitable for *Rhododendron* identification [14]. Currently, six ISSR primers, i.e., UBC826, UBC835, UBC836, UBC840, UBC890 and UBCM06, generated clear and highly polymorphic bands for the 46 rhododendron accessions. Functional tests of the selected IRAP and ISSR markers (Table 1) revealed that they had good reproducibility and resolution for the 46 accessions concerned (Figure 1). For IRAP markers, a total of 123 scorable amplified fragments (alleles) were obtained, of which 119 (96.7%) were polymorphic, with an average of 14.9 polymorphic loci generated per primer, with a size of 0.15–2.7 kb (Table 1). Among them, LTR3-21 produced the most polymorphic loci (17), while LTR1-7 and LTR1-11 generated the least (both 13). For ISSR markers, a total of 86 fragments were scored, 79 of which (91.9%) were polymorphic, and an average of 13.2 polymorphic loci were harvested per primer, with a size range of 0.2–2.6 kb. UBC836 and UBC840 generated the most polymorphic loci (both 16), whereas UBC826 generated only 10 polymorphic loci.

Among IRAP primers, LTR1–17 gave the highest Ne (1.77) and I (0.61), and LTR3–21 had the highest H (0.43) (Table 1). The average values of Ne, H and I for all IRAP primers were 1.59, 0.35 and 0.53, respectively. In terms of ISSR primers, UBC840 had the highest Ne (1.84), H (0.45) and I (0.64), and the average values of Ne, H, and I of all ISSR primers were 1.69, 0.39 and 0.57, respectively. Overall, the ISSR markers were slightly larger than the IRAP markers with respect to Ne, H and I parameters.

**Figure 2:**
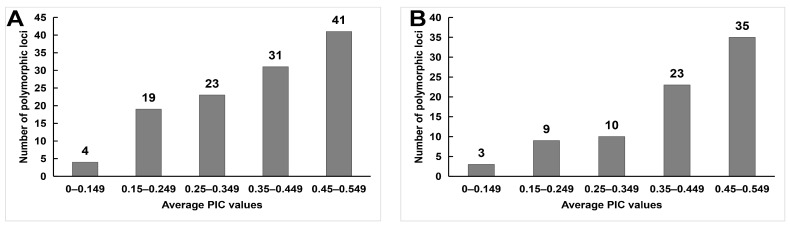
Distribution of average PICs from IRAP (**A**) and ISSR (**B**) markers among 46 rhododendron accessions.

**Figure 3:**
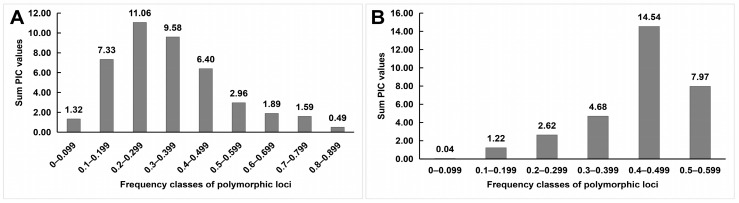
The relationship between the frequency of polymorphic loci and the total PIC value, which was amplified by IRAP (**A**) and ISSR (**B**) markers in 46 rhododendron accessions.

The mean PIC for IRAP markers ranged from 0.294 (LTR1–15) to 0.425 (LTR3–21), with an overall mean of 0.35 (Table 1). Most of the polymorphic loci of IRAP markers had PIC values ranging from 0.45 to 0.549 (Figure 2A). The mean PIC of ISSR primers was close to that of IRAP primers, 0.388, ranging from 0.326 (M06) to 0.448 (UBC840). Moreover, the PIC values of the polymorphic loci of most ISSR markers were also located at 0.45–0.549 (Figure 2B). To address the question of how the sum of PICs relates to the frequency of polymorphic loci, we analyzed the correlation between the two parameters (Figure 3). For the IRAP markers, the most informative markers (total PIC value was 11.06) were located in the frequency class of polymorphic 0.200–0.299 loci, followed by the frequency category of 0.3 to 0.399. Among the ISSR loci, polymorphic loci with frequency classes 0.400–0.499 were the most informative, followed by frequency classes 0.500–0.599.

Among the IRAP markers, the highest EMR value was from LTR3-21 (17) and the lowest was from LTR1–7 (12) (Table 1). Meanwhile, primer LTR1-17 had the highest Rp value (11.07) and MI value (7.23), while the lowest values came from LTR1–16 (6.18 and 4.11, respectively). The largest EMRs among ISSR primers were generated at UBC836 and UBC840 (both 16). Additionally, the largest Rp and MI were generated in UBC840 (12 and 7.15, respectively). Interestingly, the average Rp of ISSR markers was slightly greater than that of IRAPs, but the EMR and MI of IRAPs were greater than those of ISSRs.

### 2.3. Detection of Genetic Diversity among Rhododendron Accessions

**Figure 4:**
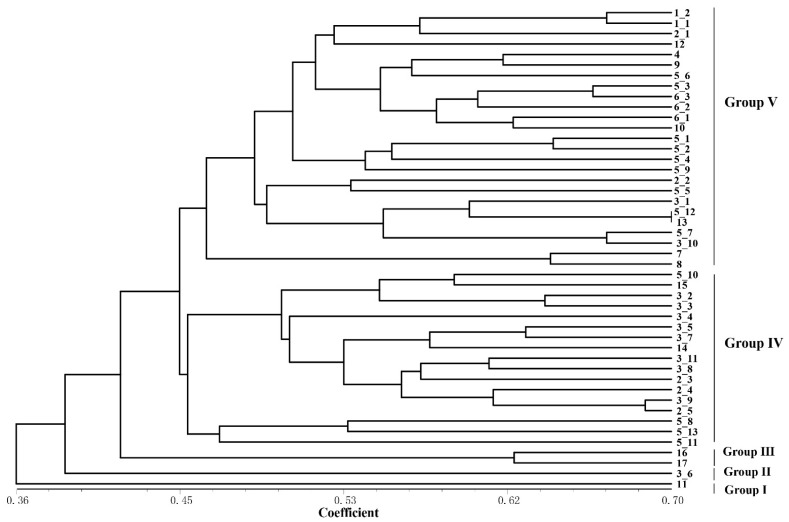
The genetic relationship dendrogram of IRAPs amplification from 46 rhododendron accessions constructed using the UPGMA method based on the Jaccard similarity coefficient. To assess the robustness of each node, a bootstrap with 1000 replicates was performed on the loci data.

**Figure 5:**
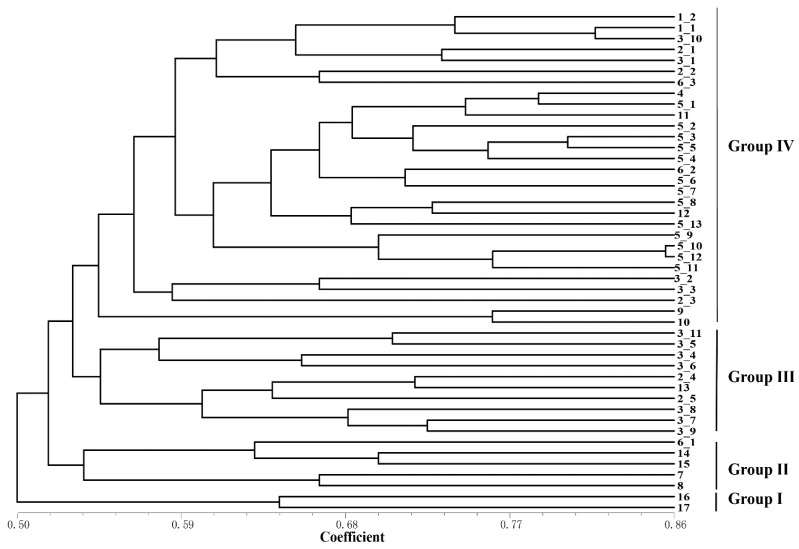
The genetic relationship dendrogram of ISSR amplification from 46 rhododendron accessions constructed using the UPGMA method based on the Jaccard similarity coefficient. To assess the robustness of each node, a bootstrap with 1000 replicates was performed on the loci data.

**Figure 6:**
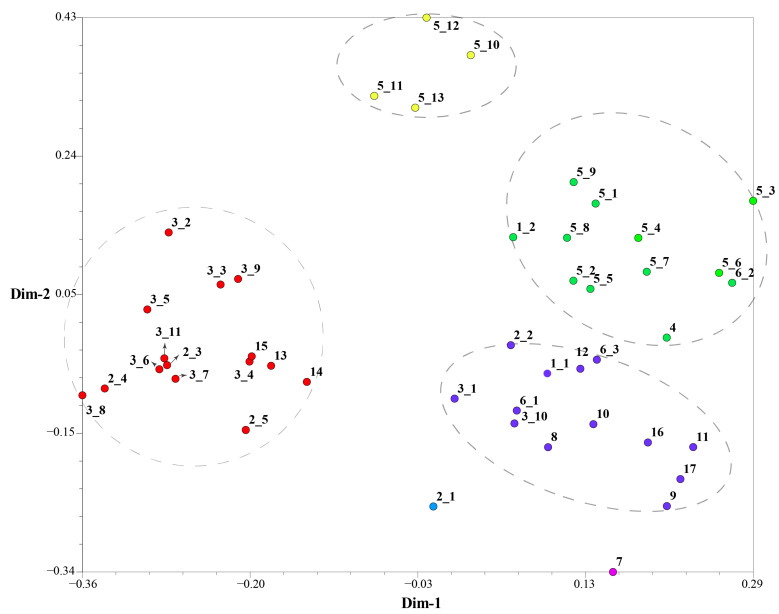
Integrated two-dimensional plot of principal coordinate analysis (PCoA) based on ISSRs and IRAPs of 46 rhododendron accessions. Similar colors represent related groups.

Based on the detection results of IRAP and ISSR markers, the UPGMA clustering algorithm was used to cluster 17 *Rhododendron* species (46 accessions), which can be divided into five and four clusters, respectively (Figure 4 and Figure 5). The dendrogram plotted by the IRAP markers showed that accessions 11 and 3_6 fell into two distinct clusters (respectively Group I and Group II). Accessions 16 and 17 were also split into a separate group (Group III). The other accessions were grouped into two further subclusters (Group IV and Group V). In the dendrogram plotted by ISSR markers, Group IV contained the most (29), followed by Group III (10), Group II (5) and Group I (2). Because ISSRs and IRAPs represent different genomic components (repeated sequences and retrotransposons, respectively), we merged the two sets of data, and finally six groups were obtained after superclustering. This joint analysis resulted in the separation of the two accessions 2_1 and 7 separately (Figure 6).

The accessions 2_1 and 7 are far apart from another group of *Rhododendron decorum* (5_10–5_13). *R. decorum* and others (5_1–5_9) did not gather together, indicating that their genomes might have mutated. Interestingly, the two separated *R. decorum* clusters also belonged to two populations (Table S1), suggesting that geographical distances may have contributed to intraspecies differences. Furthermore, accessions 3_1 and 3_10 (*R. delavayi*) were not found to cluster with any other *R. delavayi* (accessions 3_2 and 3_3, etc.), which also showed that their genomes were different from other *R. delavayi* accessions. The three groups with the largest number of accessions (the red dots, the purple dots and the green dots) clustered 15, 13 and 12 rhododendron accessions, respectively. In summary, the combinatorial clustering format with the combination of the two marker systems was expected to be more discriminative than the single procedure.

For the three populations involved in this study (*R. delavayi*, *R. decorum* and *R. agastum*), *R. decorum* demonstrated the highest Ne (1.38), H (0.25) and I (0.39) (Table 2), which may indicate that the population diversity of *R. decorum* should be the most abundant in this area, partially reflecting the better adaptation of *R. decorum* to the environment in BDNR of Guizhou Province, China.

### 2.4. Genetic Fidelity Assessment of In Vitro Cultures of the Endangered R. bailiense

**Figure 7:**
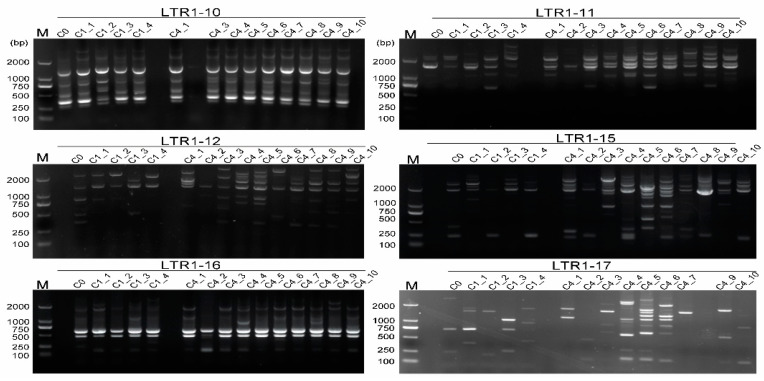
Detection of genetic fidelity of *R. bailiense* calli in somatic clones using IRAP markers. The lanes corresponding to the subclone codes are marked on the top of the gel map. Among them, C0 stands for the primary cycle; C1_1-C1_4 represent the callus from the first cycle of subculture; C4_1–C4_10 represent the materials after the forth cycle of subculture. Lanes that did not exhibit bands were retested and the binary data obtained were incorporated into subsequent analyses. M, D2000 DNA marker (Tiangen Biological Company, Beijing, China).

Based on the IRAP and ISSR profiles from the 46 rhododendron accessions, IRAP and ISSR markers were employed to evaluate genetic variation of different callus-subcultured cycles of *R. bailiense*, an endangered species only found in BDNR. The fingerprints yielded from six IRAP primers and six ISSR primers with the most polymorphic loci demonstrated that they were effective for somaclonal variation detection (Table 3). In *R. bailiense*, IRAPs were effective at detecting genetic fidelity from in vitro cultures (Figure 7). Among them, some primers, e.g., LTR1–15 and LTR1–17, produced more polymorphic bands. In terms of ISSRs, the six ISSR primers were also effective at detecting the genetic fidelity of *R. bailiense* during subculture (Figure S6), however, ISSRs were obviously less effective than IRAPs in somaclonal variation assessment since the former detected far fewer polymorphic fragments (Figure 7 and Figure S6, Table 3).

**Figure 8:**
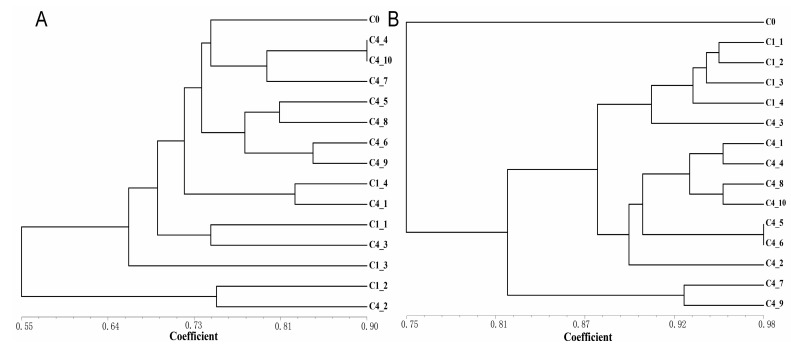
The UPGMA dendrogram of somatic clones derived from *R. bailiense* calli as revealed by IRAP (**A**) and ISSR (**B**) markers. To assess the robustness of each node, a bootstrap with 1000 replicates was performed on the data on loci.

The Jaccard similarity coefficients between the calli of different subculture cycles were calculated based on the results of marker detection, and the UPGMA cluster analysis was carried out (Figure 8). Generally, the most similarity in IRAPs was investigated among the subclones within the same cycle, and higher genetic aberration was observed beyond the cycles (Figure 8A). Within the ISSRs detection, the four subclones of C1 and C4_3 were clustered into the same group (Figure 8B), and the remaining C4 subclones gathered into another. The similarity coefficients generated by ISSRs were generally larger than those generated by IRAPs, indicating that IRAPs are more effective in somaclonal variation detection, which at least partially reflected that the cause of somaclonal variation of *R. bailiense* was ascribed to the activation of retrotransposons.

## 3. Discussion

Transposons make up a large proportion of plant genomes. In *Rhododendron*, repetitive elements account for over a half (57.00%) of the genome, and LTR retrotransposons consist of the largest part, reaching 24.76% [1]. The difference between *Copia* and *Gypsy* is not only reflected in the gene structure, but also in the copy number, insertion time, characteristics and influence on the genome [15]. In the current case, we gained seven IRAP primers designed with *Copia* sequences and one IRAP primer designed with *Gypsy* sequence, and their average PIC value was 0.36. The six highly informative ISSR primers tested herein yielded an average of 13.2 polymorphic loci per primer (Table 1). The combination of two marker systems was proven to be effective for genetic diversity evaluation and somaclonal variation detection in *Rhododendron* germplasm.

### 3.1. IRAPs and Its Effectiveness in Genetic Divergence Detection for Rhododendron Germplasm

Previously, the ISSR marker system was justified to be suitable for the identification of genetic diversity in rhododendron as well as the detection of genetic fidelity in somatic clone reproduction [14]. In the present study, an IRAP marker was first developed in *Rhododendron* plants based on the sequences of retrotransposons. To evaluate the effectiveness of the two marker systems, a comparison between ISSR and IRAP markers was carried out since they represent different genome components.

The IRAP markers represent LTR retrotransposons and the ISSR markers represent repeat sequences. Although the areas they cover may overlap, there exists a remarkable difference. The two markers based on different sequence features may be an intriguing and worthy direction to explore the characteristic parameters they exhibit. The PICs of IRAP (0.36) and ISSR (0.38) were not much different, and the number of polymorphic bands was also close, being 14.9 and 13.2, respectively. The IRAP markers showed a high degree of polymorphism in *Rhododendron* plants, as high as 96.7%, which is close to that of 94% polymorphisms in *Bletilla striata* [16], and 98% polymorphisms in *Phyllostachys* species [13] as calculated by IRAPs. Previously, ISSRs were demonstrated to be polymorphic for genetic variation detection [8]; the IRAP marker is now shown to be more effective in polymorphism assessment for rhododendrons. Likewise, EMR is the number of markers generated per primer assay [17], and the EMR value for IRAPs (14.55) was greater than that for ISSRs (12.21). Sheik et al. (2019) analyzed the polymorphism of 24 rhododendron accessions using 14 microsatellite markers, and scored an average of 2.92 alleles per primer [18]. The marker index (MI), which evaluates the efficiency of markers to detect polymorphisms, was also about 10% higher for IRAPs (5.23) than for ISSRs (4.79). Markers with higher MI values are the preferred markers in plant species identification and plant DNA fingerprinting [17]. Therefore, the IRAP marker system developed herein is considerably superior at genetic diversity detection to the highly informative ISSRs as identified previously.

As in sugarcane (*Saccharum* spp.), IRAPs and ISSRs exhibit different characteristics in species clustering analysis [17]; a similar tendency was investigated in the rhododendrons (Figure 4 and Figure 5). For example, the coefficient of the IRAPs was 0.36–0.7, while that of ISSRs was 0.5–0.86, showing that IRAPs were somehow more sensitive at detecting genetic differences in rhododendrons. The IRAPs could divide the 46 rhododendron accessions into five groups, whereas the ISSRs divided them into four groups. Interestingly, some accessions from different populations of the same species were clustered into separate groups, e.g., accessions 3_1, 3_6 and 3_10 of *R. delavayi*, as revealed by IRAP markers. In ISSR-marker-associated clustering, seven accessions of *R. delavayi* were classified in Group III and another four were clustered in Group IV. The results mentioned above also reflect that IRAPs were more effective at detecting genetic difference in rhododendrons. Additionally, a comparatively high polymorphism was investigated by IRAPs among the accessions from different populations of *R. delavayi* distributed within 2 km of the Nature Reserve, indicating that high divergences may exist among the different populations of this species. This could possibly be ascribed to the occurrence of retrotransposons, finally leading to better adaptation to the environment in BDNR. The above results also indicate that IRAP markers can easily identify genomic variation within *Rhododendron* species.

### 3.2. A Combination of IRAPs and ISSRs Shows More Effectiveness in Genetic Diversity Assessment in Rhododendrons

Considering the differences of IRAP and ISSR markers, we investigated the combination of IRAPs and ISSRs so as to evaluate the clustering effect among the rhododendron accessions. The IRAP marker system for rhododendrons was developed herein based on retrotransposon sequences, and ISSRs were from repeat sequences. Interestingly, the clustering efficiency of the combined two markers was better than that of a single one, and the accessions from *R. delavayi* were more likely to cluster together in comparison with those identified by one marker system.

In exception of the seven accessions from *R. delavayi*, two accessions 3_1 and 3_10 were clustered into another category. In fact, these two accessions were geographically apart from the others by about one kilometer, possibly leading to subtle genetic differences from the others, which could be more effectively evaluated by the combination of the two marker systems. Additionally, in *R. decorum*, four accessions, i.e., 5_10–5_13, were grouped separately. Geographically, these four accessions were separated from the other population (accessions 5_1–5_9) by approximately two kilometers, where the topographical characters were somewhat different, leading to differences in rainfall and temperature, which might promote transposition events so as to enhance adaptive evolution. Most interestingly, rhododendrons that were located closer together were more likely to cluster together as revealed by the combination of the two marker systems, even though they were different species. For example, the accessions 13, 14 and 15 demonstrated a very close genetic relationship. This may be due to the proximity in geographic location, which can easily lead to cross pollination between species, resulting in the mutual merging of genomes between offspring. Overall, the combination of the IRAP markers and the ISSR markers appears be able to better reveal the synteny and divergence among species.

### 3.3. Somaclonal Variation as Detected by IRAPs and ISSRs

The use of ISSRs to detect the genetic fidelity of somaclonal clones has been reported in *Rhododendron* [8]. In this study, IRAPs and ISSRs were employed to detect the somaclonal variation among the 15 individuals of *R. bailiense* calli which were subcultured for different cycles. Both could effectively distinguish some somaclonal variation in the *R. bailiense* genome (Figure 7 and Figure S6). However, the two markers showed different detection properties. This is primarily due to certain IRAP markers that can facilitate the discovery of variants in the somatic clones of *Rhododendron*, such as LTR1–11, LTR1–12 and LTR1–15 (Figure 7). Interestingly, some IRAP primers used herein, e.g., LTR1–11, LTR1–12 and LTR1–15, detected a large number of polymorphic bands during in vitro culture, which might be ascribed to the occurrences in transposon events activated by in vitro conditions. Similar results have also been documented in rice [19] and sugarcane [20], in which the transposon was strongly activated under tissue culture conditions. Meanwhile, the above findings also suggest that retrotransposons from *Copia*, e.g., TY1–30, TY1–33 and TY1–42, which correspond to IRAP primers LTR1–11, LTR1–12 and LTR1–15, might be the active transposons in *R. bailiense*, which are easily activated by the external factors to trigger mutations in the genome.

Compared with IRAPs, the variation detected by ISSRs was significantly less (Figure 7, Figure 8 and Figure S6), which was reflected by the higher similarity coefficient values of tissue-cultured *R. bailiense* callus as displayed by ISSR markers. It is noteworthy that the rate of genetic variation detected by ISSR markers was low (less than 5%) in some species of *Rhododendron*, such as *R.* ‘Kazimierz Odnowiciel’ [8] and *R. mucronulatum* [21], which might also be due to the difference in in vitro materials (seedlings and callus) as well as in medium formulae. The calli used herein should be more prone to variation during subculture in comparison to the seedlings [22]. Therefore, although IRAPs and ISSRs have similar PIC values, the former are obviously better suited to detect somatic variation in somatoclonal replication.

It is noteworthy that most of the transposons are also associated with repeat sequences [23], which may lead to the genetic variation as detected by ISSRs due to the activity of transposons. Evidences in sugarcane also showed that polymorphic fragments amplified by the ISSR markers might be transposable element sequences generated by transposon activity [17]. Therefore, somaclonal variation detected by ISSR markers may also be related to transposon events.

In conclusion, the IRAP markers developed in the present study provide a novel methodology to detect somaclonal variation in *Rhododendron* tissue culture. The in vitro culture used herein was callus, which is generally prone to genetic instability, and thus demonstrated high frequency in somaclonal variation. Our preliminary investigation in plantlets of *Rhododendron* species also showed that IRAPs can also detect genetic variation, and a systematic study should be further carried out for in vitro plantlet detection so as to facilitate the preservation of *Rhododendron* germplasms.

## 4. Materials and Methods

### 4.1. Plant Material

In total, 46 accessions of wild rhododendrons (Table S1) were collected from the BDNR (27.22° N, 105.84° E) (Figure S1) from Bijie City, Guizhou Province, China, and the sampling materials were young leaves in May 2022. Once the samples were collected, they were stored in cryopreservation tubes, placed in liquid nitrogen, transported back to the laboratory and then stored in a refrigerator at −80 °C for long term storage.

Pods of *R. bailiense* containing seeds were collected from the adult trees and brought back to laboratory for seed extraction. The seeds were first completely dried and then washed with 75% alcohol (Fengchuan Chemical Reagent Technology Co., Ltd., Tianjing, China) for 30 s [14]. The seeds were then soaked in 10% sodium hypochlorite (Jiangchuan Chemical Group Co., Ltd., Chongqing, China) for approximately 15 min, followed by 5 washes with sterile distilled water. For germination, disinfected seeds were plated on WPM basal medium (Baisi Biotechnology Co., Ltd., Hangzhou, China) + 1.0 mg·L^−1^ kinetin (KT, Meilun Biological Technology Co., Ltd., Dalian, China) [14]. In addition, 3% (*w*/*v*) sucrose (Fengchuan Chemical Reagent Technology Co., Ltd., Tianjing, China) and 0.7% (*w*/*v*) agar (Solarbio Science & Technology Co., Ltd., Beijing, China) were added to the medium and adjusted to pH 5.5, which was autoclaved at 121 °C for 20 min. The seeds germinated in a culture room with a 14 h photoperiod, supplied with bright white-LED light with a light flux density of 50 μmol m^−2^ s^−1^ and a room temperature of 25 °C. At least ten glass bottles with a diameter of 7 cm filled with germination medium were used to germinate *R. bailiense* seeds. After 45–60 days, *R. bailiense* seedlings were transferred to callus induction medium (WPM + 0.2 mg·L^−1^ thidiazuron <TDZ, Meilun Biological Technology Co., Ltd., Dalian, China> + 0.1 mg·L^−1^ 3-indole butyric acid <IBA, Boao Tuoda Technology Co., Ltd., Beijing, China>) to induce callus [24]. Approximately 60 days later, the cultured calli (Figure S5) were transferred to the proliferation medium (WPM + 1.0 mg·L^−1^ 2,4-dichlorophenoxyacetic acid <2,4-D, Solarbio Science & Technology Co., Ltd., Beijing, China> + 0.5 mg·L^−1^ 1-naphthalcetic acid <NAA, Solarbio Science & Technology Co., Ltd., Beijing, China>) for proliferation [25]. Each individual callus was numbered, and the callus corresponding to each digit proliferated independently. At last we obtained at least ten groups of calli from each of the ten previously grown *R. bailiense* seedlings. Therefore, their respective genomes should be relatively stable. Three groups of callus material in a good growth state were selected and subcultured approximately every 40–50 days (still using the proliferation medium).

### 4.2. DNA Extraction

DNA in fresh rhododendron leaves was extracted using Plant Genomic DNA Extraction Kit (Tiangen Biochemical Technology Co., Ltd., Beijing, China). A Kaiao K5500 spectrophotometer (Kaiao, Beijing, China) was employed to measure the OD value of DNA at 260 nm, and calculated its purity and concentration. The quality was checked by 1% agarose (Biowest, Nuaillé, France) gel electrophoresis. The gel was observed and photographed with a gel imaging system Quantum CX5 Edge 18.02a (Vilber Lourmat, Marne-la-Vallée, France). Qualified DNA samples were stored at −20 °C for later use.

### 4.3. LTR Retrotransposon Cloning and Sequencing

The reverse transcriptase (RT) domain of the LTR retrotransposon (*Copia* and *Gypsy* superfamily) in *Rhododendron* was cloned using the leaves of *R. delavayi*, and the primers were *Copia*, *Copia1*: 5′-ACNGCNTTYYTNCAYGG-3′ and *Copia2*: 5′-ARCATRTCRTCNACRTA-3′ [26]; *Gypsy*, *Gypsy1*: 5′-AGMGRTATGTGYGTSGAYTAT-3′ and *Gypsy2*: 5′-CAMCCMRAAMWCACAMTT-3′ [27] (Synthesized by Sangong Biotech, Shanghai, China). The PCR amplification volume was 50 µL, of which 25 µL 2 × *Taq* PCR Mastermix (Tiangen Biochemical Technology Co., Ltd., Beijing, China) (containing 0.1 U/µL *Taq* polymerase, 500 µM dNTP, 20 mM Tris-HCl, 100 mM KCl and 3 mM MgCl_2_, pH8.3), 20 ng genomic DNA of *R. delavayi* leaf, 0.5 µM of forward primer and reverse primer each, and ddH_2_O to constitute the volume of the reaction. The reason why *R. delavayi* leaf was chosen as the object of LTR retrotransposon cloning was mainly because it has a draft genome assembly [28], which may be beneficial to future studies on the insertion sites of LTR retrotransposons and their impact. In addition, *R. delavayi* is also the most widely distributed *Rhododendron* species in the BDNR. The PCR reaction program included pre-denaturation at 94 °C for 3 min; 35 cycles of denaturation at 94 °C for 1 min, annealing for 1 min (TY1-*Copia*: 53 °C; TY3-*Gypsy*: 50 °C), extension at 72 °C for 1.5 min, and final extension at 72 °C for 5 min. The PCR-amplified products were electrophoresed at 150 V for 30 min in 2% agarose gel containing GelRed (BBI Life Sciences Corporation, Shanghai, China), and then observed and photographed in the gel imaging system Quantum CX5 Edge 18.02a. We cut and separated the target DNA bands, and then purified the resulting DNA fragments using TakaRa MiniBEST Agarose Gel DNA Extraction Kit Ver. 4.0 (TaKaRa, Kyoto, Japan). The PCR-amplified product was ligated into the pEASY-T1 Cloning Vector (Transgene Co., Ltd., Beijing, China). The fusion vector was transformed into *Escherichia coli* (*E. coli*) strain DH5α (Tiangen Biochemical Technology Co., Ltd., Beijing, China). After culture on the plate, a single clone was picked for sequencing. The sequence generated by the sequencing was the RT domain of the LTR retrotransposon. Recombinant white clones were screened by PCR amplification (using M13F and M13R primers), after which plasmids were isolated and sequenced (Sangon Biotech, Shanghai, China) using the universal primers M13F and M13R. Each generated sequence was the RT domain sequence of an LTR retrotransposon. The obtained sequences underwent multiple sequence alignments and the evolutionary distance was calculated using the maximum likelihood method with 1000 bootstraps to construct a phylogenetic tree by employing ClustalW in the MEGA7 software (version 7.0.14) [29].

### 4.4. Design of IRAP Primer

IRAP primers were designed according to the conserved sequences of the RT domains of TY1-*Copia* and TY3-*Gypsy* (Table 2). Primers were synthesized by Sangon Biotech (Shanghai, China). Genomic DNA from leaves of *R. delavayi* was selected as template DNA, and the reaction system was (10 µL): 10–50 ng of template DNA, 1 µL of primers (10 µM), 5 µL 2 × *Taq* PCR Mastermix and the appropriate amount of ddH_2_O. The PCR reaction procedure was similar to that described above (see the Section 4.3), except that the annealing temperature was set as a gradient (55–60 °C) to screen the optimal annealing temperature for each IRAP primer. The PCR amplification product was electrophoresed at 150 V for 50 min in 2% agarose gel containing GelRed, and the gel was then observed and photographed in the gel imaging system.

### 4.5. Polymorphism Detection of Wild Rhododendron Accessions

For the dozens of rhododendron accessions for polymorphism detection (Table S1), a total of 8 IRAP primers and 6 polymorphic ISSR primers were used (Table 3). The reaction system for IRAP amplification and ISSR amplification was the same as described above (see the Section 4.3), except that the annealing temperature was adjusted for each primer (Table 1). The PCR amplification product was electrophoresed at 150 V for 50 min in 2% agarose gel containing GelRed, and then the gel was observed and photographed in the gel imaging system.

### 4.6. Genetic Fidelity Assessment for In Vitro Cultures

To detect genetic fidelity of in-vitro-grown *R. bailiense*, a group of calli with the best proliferation was collected at the primary (C0, one clone), the first (C1, four clones), and the fourth (C4, 10 clones) cycles. About 0.1 g of callus was isolated to extract DNA, which was used for IRAP and ISSR amplification to assess the genetic fidelity.

### 4.7. Data Analysis

PCR amplification of each ISSR and IRAP primer involved in this study was performed in at least three replicates. The GelQuest software (version 3.5.5.0) (https://www.sequentix.de/gelquest/, accessed on 23 January 2023) was employed for statistics, and only clear bands of ISSR or IRAP were counted as “1” (presence); conversely, absences were recorded as “0”. Low-intensity bands (difficult to distinguish presence or absence) were not considered in this step.

We used POPGENE1.32 software [30], as subjected to Hardy–Weinberger balance, to compute the average number of Shannon’s information index (I), Nei’s genetic diversity index (H) and effective number of alleles (Ne).

To compare the suitability of the IRAP and ISSR markers, the information content and level of polymorphism were assessed with several parameters including resolving power (Rp), effective multiplex ratio (EMR), marker index (MI), polymorphic information content (PIC) and mean of PIC [31].

The PIC index for each loci was calculated using the following formula [32]:

PIC_i_ = 2f_i_ (1 − f_i_),

where PIC_i_ represents the PIC value of loci i, and fi represents the frequency of the amplified bands. The PIC for each primer was calculated by taking the average of the PIC values for all loci.

MI was calculated using the following formula [33]:

MI = EMR × PIC,

where EMR is the product of the proportion of the number of polymorphic loci per assay and the total number of polymorphic loci.

The Rp value of each primer was calculated with the following formula [34]:

RP = ∑Ib,

where Ib represents the fragment that provides information. This parameter can be calculated with the following formula:

Ib = 1 − [2 × (0.5 − p)],

where p represents the proportion of individuals that contain a band.

### 4.8. Cluster Analysis

Numerical Taxonomy System (NTSYS-pc) ver. 2.10e was used to construct dendrograms and similarity matrices [17]. The Jaccard’s similarity coefficient between each pair of accessions was then calculated using the binary data matrix via the SIMQUAL model [17]. The above distance coefficients were employed to construct a dendrogram through the unweighted grouping method arithmetic mean method (UPGMA), and the Dice similarity coefficient algorithm was applied to determine the genetic diversity among accessions. To highlight the classification resolution, we performed principal coordinates analysis (PCoA) using the EIGEN module of NTSYS-pc. Once the PCoA cluster diagram was generated, Adobe Illustrator CS5 (https://www.adobe.com/products/illustrator.html, accessed on 10 February 2023) was used to embellish the image.

## 5. Conclusions

In conclusion, we developed the IRAP marker system in *Rhododendron*, and examined both IRAPs and ISSRs as possible candidates with the goal of establishing a reliable system for identifying genetic fidelity and diversity. They were found to have complementary effects when testing the genetic diversity of multiple *Rhododendron* species and the genetic fidelity of *R. bailiense* somaclonal clones. Particularly, IRAPs were superior to ISSRs in the number of polymorphic bands, EMR and MI, and were more suitable for detecting the somatic variations of *R. bailiense*. Future work may focus on unraveling the causes and mechanisms of this genetic variation. Moreover, it is also essential to explore how to improve the genetic fidelity of rhododendron germplasm preserved in vitro.

## Figures and Tables

**Table 1:** Information content of IRAP and ISSR markers and other polymorphism indices from 46 wild rhododendron accessions.

	Ne	H	I	Number of Polymorphic Alleles	Range of Alleles (kb)	PIC	EMR	Rp	MI	Annealing Temperature (°C)
IRAP										
LTR1–7	1.62	0.37	0.55	13	0.2–2.3	0.37	12.07	6.98	4.41	56
LTR1–10	1.54	0.33	0.51	15	0.3–2.7	0.33	15.00	7.20	5.01	59
LTR1–11	1.64	0.37	0.56	13	0.4–2.4	0.37	14.00	7.07	5.25	59
LTR1–12	1.47	0.30	0.47	16	0.3–2.9	0.30	16.00	6.38	4.83	59
LTR1–15	1.51	0.32	0.49	15	0.2–2.4	0.32	13.24	6.70	4.20	55
LTR1–16	1.51	0.31	0.48	14	0.15–2.6	0.31	13.07	6.18	4.11	55
LTR1–17	1.77	0.42	0.61	16	0.2–2.5	0.42	16.00	11.07	6.78	56
LTR3–21	1.76	0.43	0.61	17	0.2–2.5	0.43	17.00	11.00	7.23	57
Mean	1.60	0.36	0.53	14.88	-	0.36	14.55	7.82	5.23	57
ISSR										
UBC826	1.79	0.43	0.62	10	0.3–2.0	0.43	8.33	7.16	3.61	59
UBC835	1.72	0.41	0.60	15	0.3–2.4	0.41	15.00	9.14	6.20	59
UBC836	1.69	0.39	0.58	16	0.2–2.6	0.38	16.00	9.54	6.05	59
UBC840	1.83	0.45	0.64	16	0.2–2.3	0.45	16.00	12.00	7.15	59
UBC890	1.59	0.34	0.51	11	0.3–2.3	0.34	9.31	6.18	3.18	59
UBCM06	1.48	0.30	0.46	11	0.2–2.2	0.30	8.64	4.75	2.55	59
Mean	1.69	0.39	0.57	13.17	-	0.38	12.21	8.13	4.79	59

Ne, effective allele number; H, Nei’s gene diversity; I, Shannon’s Information index; PIC, the average polymorphic information content of each loci; EMR, effective multiplex ratio; Rp, resolution power; MI, marker index.

**Table 2:** Genetic variation information of three *Rhododendron* populations.

Populations	Sample Size	Ne	H	I
*R. delavayi*	11	1.32	0.21	0.33
*R. decorum*	13	1.38	0.25	0.39
*R. agastum*	5	1.35	0.22	0.33

Ne, effective allele number; H, Nei’s gene diversity; I, Shannon’s Information index.

**Table 3:** IRAPs and ISSRs information selected to detect the genetic fidelity of *R. bailiense* callus.

Primer ID	Primer Sequence (5′–3′)	Number of Scorable Bands	Number of Polymorphic Band
IRAP			
LTR1-10	ATGGTCTTAAGCAGTCACCTC	8	2
LTR1-11	GGGCTGAAACAGTCTCCAAGA	7	6
LTR1-12	CTCCCAGACAGTGGTACAGAA	8	6
LTR1-15	CCCAGACAGTGGTACAGAAAG	7	7
LTR1-16	ACAGGCTCCCAGACAGTGGTA	6	2
LTR1-17	GAGTCTGTATGGTTTGAAACA	9	9
ISSR			
UBC826	ACACACACACACACACC	9	7
UBC835	AGAGAGAGAGAGAGAGTC	7	4
UBC836	AGAGAGAGAGAGAGAGYA	5	3
UBC840	GAGAGAGAGAGAGAGAYT	5	2
UBC890	VHVGTGTGTGTGTGTGT	7	0
UBCM06	AGCAGCAGCAGCY	9	5

The degenerate bases in the table are as follows: Y, C/T; V, G/A/C; H, A/T/C.

## Data Availability

Not applicable.

## Supplementary Materials

The following supporting information can be downloaded at: https://www.mdpi.com/article/10.3390/ijms24086902/s1.
