# Supplementary figures and images for: IRAPs in Combination with Highly Informative ISSRs Confer Effective Potentials for Genetic Diversity and Fidelity Assessment in Rhododendron
Source: Int J Mol Sci. 2023 Apr 7;24(8):6902. doi: 10.3390/ijms24086902 (PMC10138525; doi:10.3390/ijms24086902)

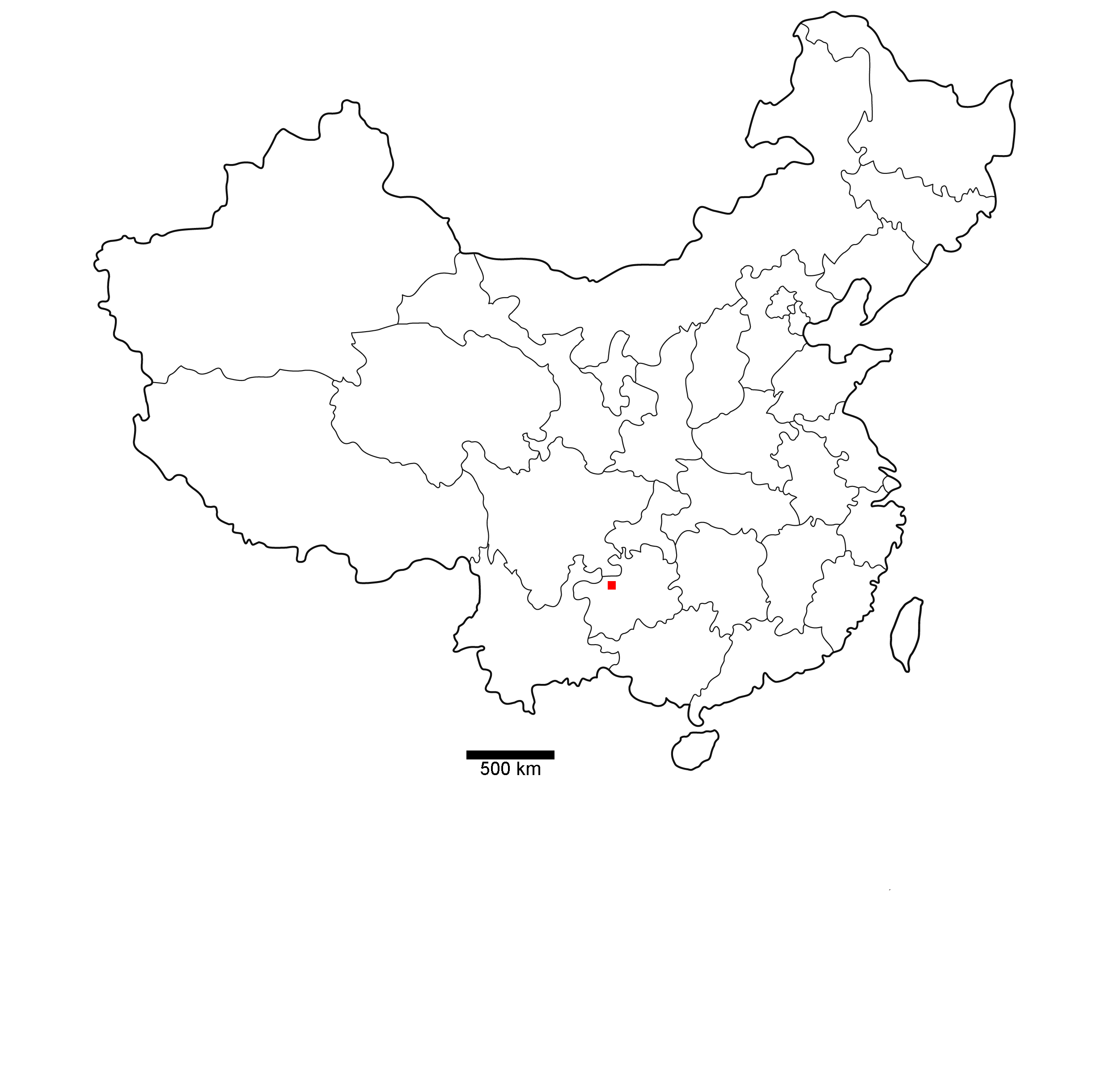

Supplement: Supplementary file 1 [file ijms-24-06902-s001.zip › S1.tif]

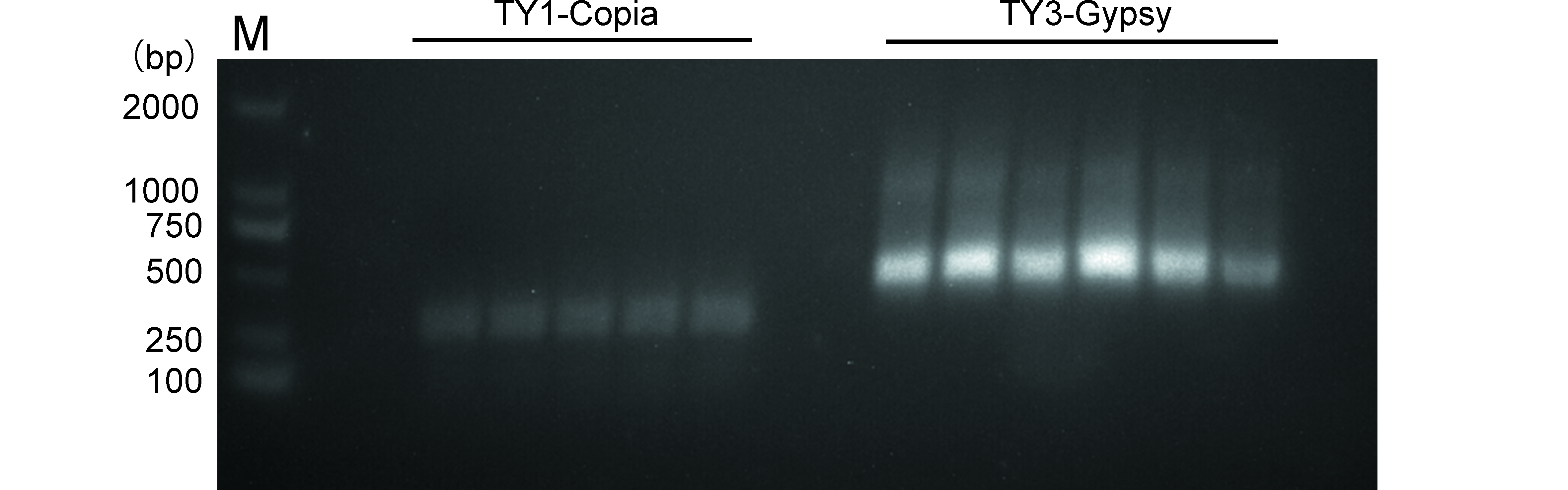

Supplement: Supplementary file 1 [file ijms-24-06902-s001.zip › S2.tif]

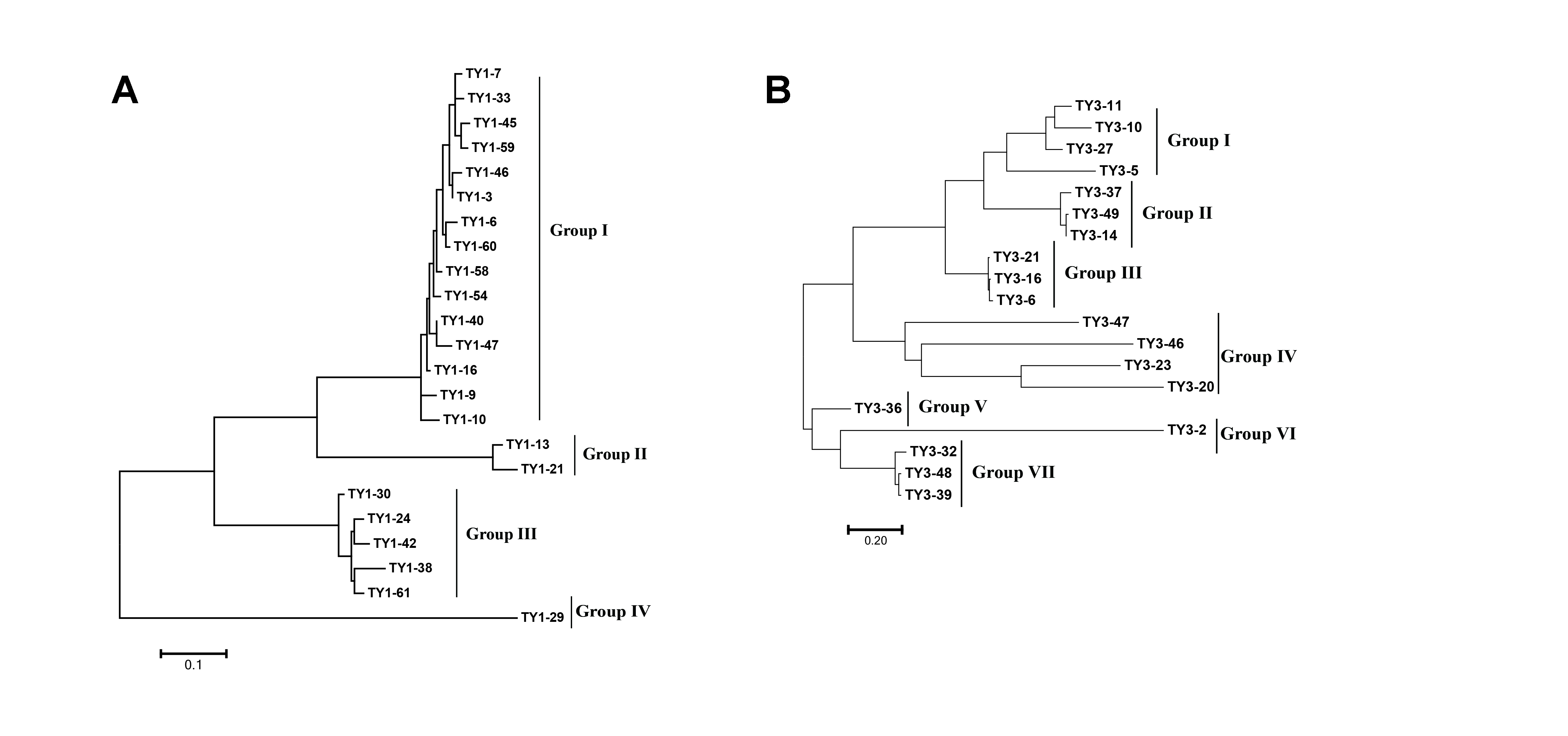

Supplement: Supplementary file 1 [file ijms-24-06902-s001.zip › S3.tif]

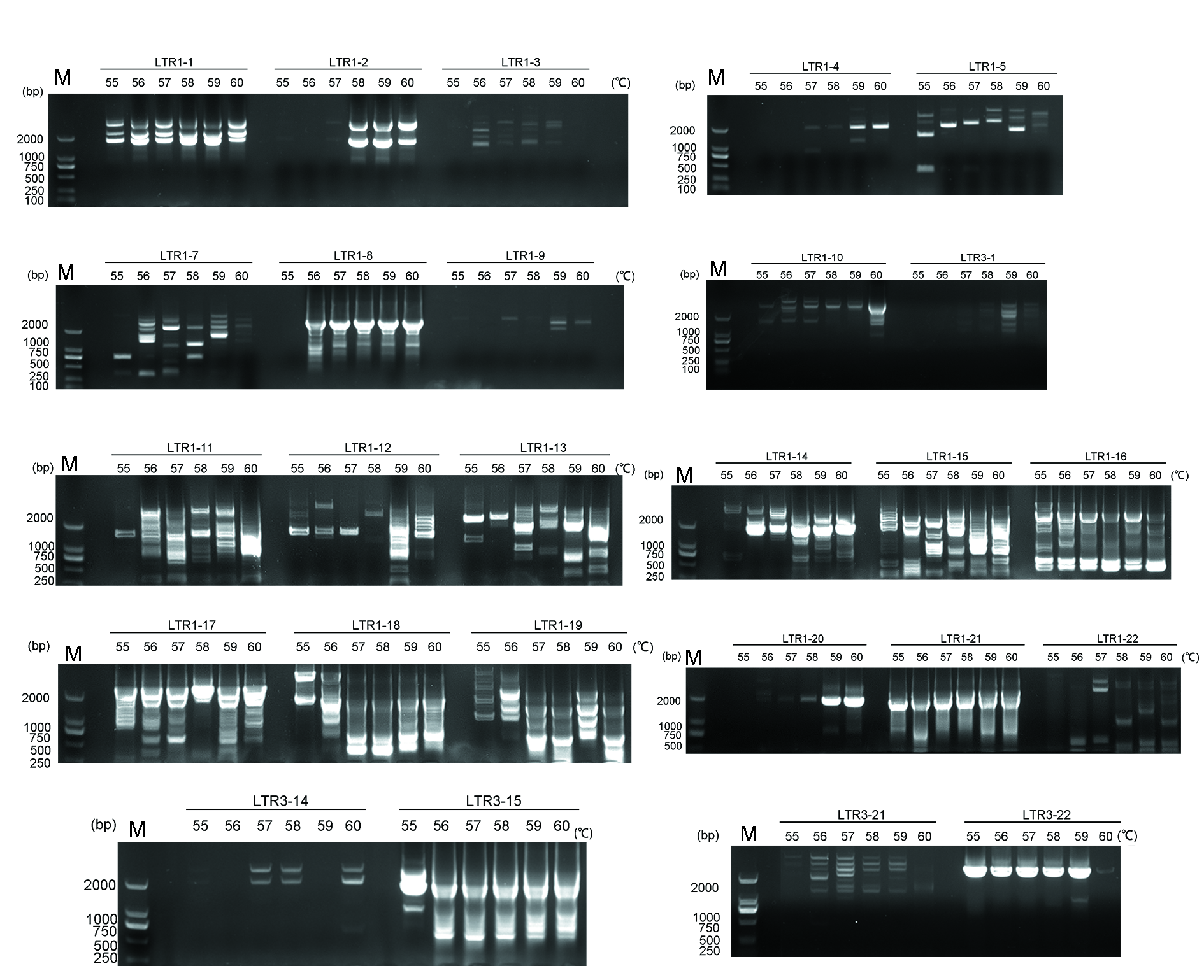

Supplement: Supplementary file 1 [file ijms-24-06902-s001.zip › S4.tif]

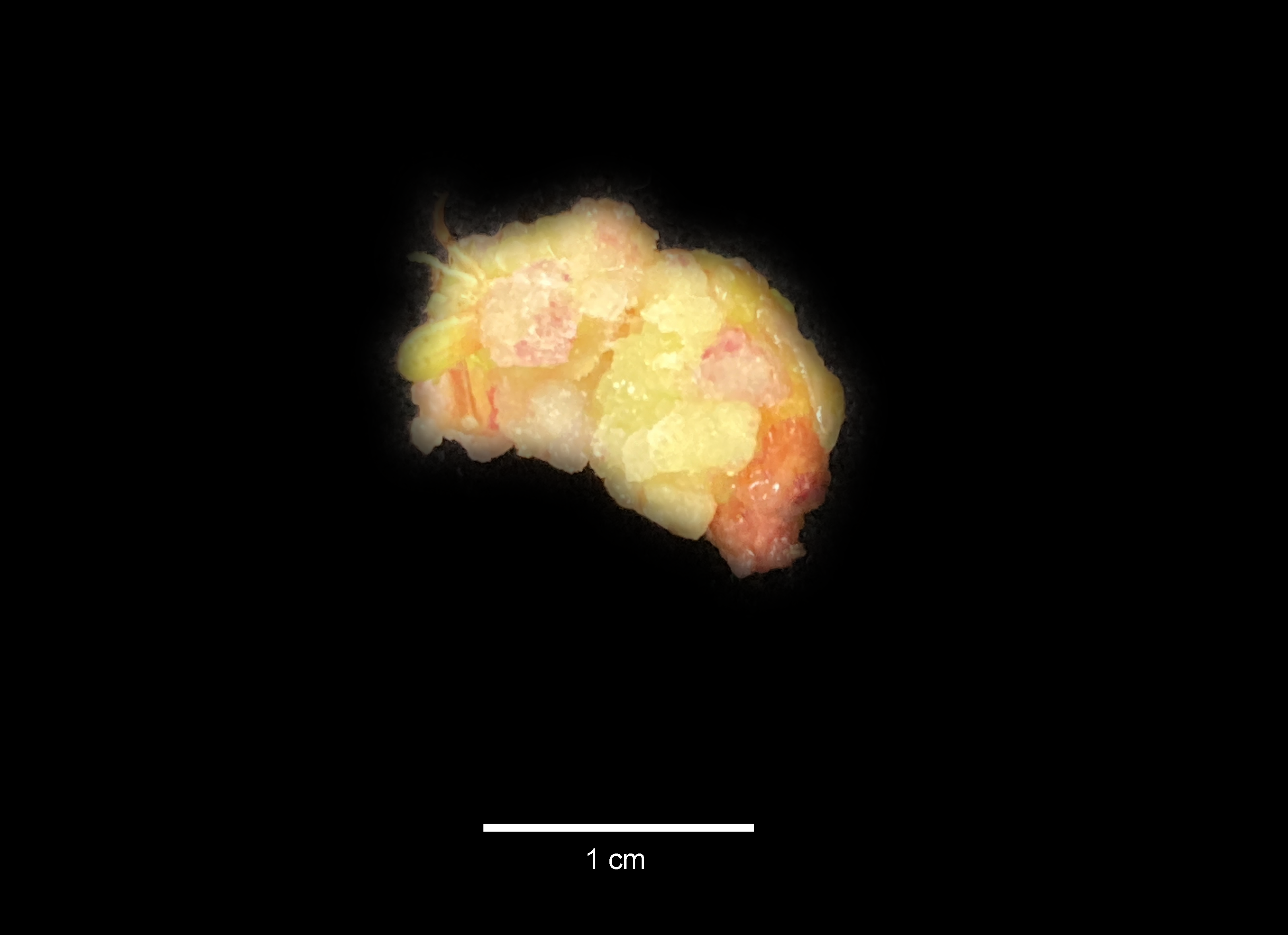

Supplement: Supplementary file 1 [file ijms-24-06902-s001.zip › S5.tif]

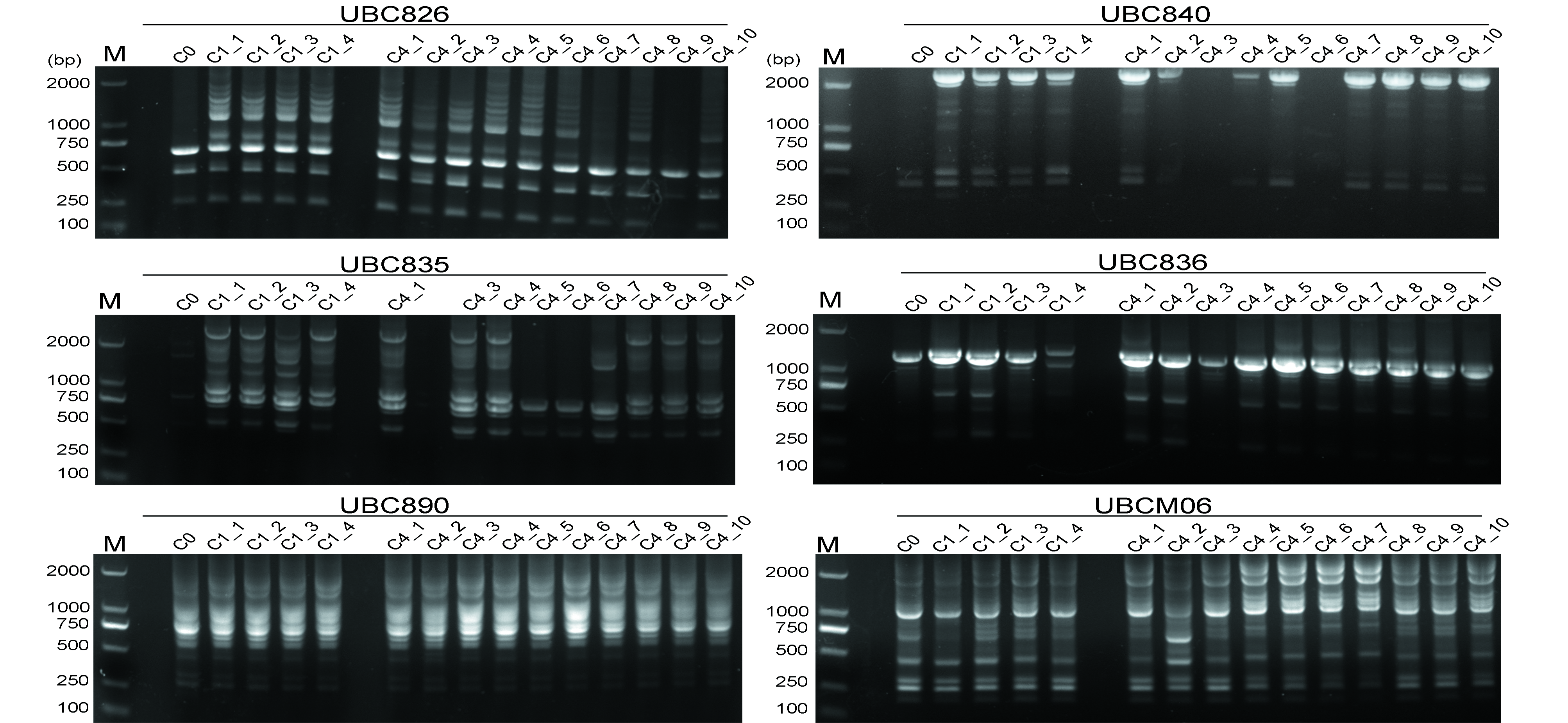

Supplement: Supplementary file 1 [file ijms-24-06902-s001.zip › S6.tif]
